# Curcumin's Protective Role in Heatstroke‐Induced Acute Liver Injury: Targeting Pyroptosis and Enhancing SIRT1 Expression

**DOI:** 10.1002/gch2.202400178

**Published:** 2024-10-13

**Authors:** Yizhan Wu, Fei Guo, Yan Ma, Weihao Chai, Jiajia Li, Wenhui Shi, Jiangwei Liu

**Affiliations:** ^1^ Department of Graduate School Xinjiang Medical University Urumqi Xinjiang Uygur Autonomous Region 830000 China; ^2^ Department of Emergency Trauma Surgery The First Affiliated Hospital of Xinjiang Medical University Urumqi Xinjiang Uygur Autonomous Region 830054 China; ^3^ Department of Anesthesiology The First Affiliated Hospital of Xinjiang Medical University Urumqi Xinjiang Uygur Autonomous Region 830054 China; ^4^ Key Laboratory of Special Environmental Medicine of Xinjiang General Hospital of Xinjiang Military Command of the PLA Urumqi Xinjiang Uygur Autonomous Region 830000 China

**Keywords:** acute liver injury, curcumin, heatstroke, pyroptosis, SIRT1

## Abstract

Heatstroke (HS) is a severe systemic condition that significantly impacts organ function, with the liver being particularly vulnerable. Sirtuin 1 (SIRT1), a crucial deacetylase, is implicated in various diseases' pathophysiology. Curcumin, a natural polyphenol, has been shown to modulate SIRT1 activity, offering therapeutic benefits. This study explores the impact of HS on hepatic SIRT1 expression and the protective mechanisms of curcumin against HS‐induced hepatic injury. Male C57BL/6 mice are divided into control and curcumin pretreatment groups, subjected to HS induction, and assessed for liver injury biomarkers, oxidative stress, and inflammatory cytokines. Results indicate that HS downregulates SIRT1, leading to liver damage and systemic inflammation. Curcumin pretreatment dose‐responsively attenuates these effects, with the highest dose providing optimal protection, potentially through SIRT1 restoration. The findings suggest that curcumin's hepatoprotective role in HS may be mediated by upregulating SIRT1, highlighting its therapeutic potential in heatstroke‐related liver damage.

## Graphical abstract

This study investigates curcumin's protective effects on heatstroke‐induced acute liver injury by targeting pyroptosis and enhancing sirtuin 1 (SIRT1) expression. The research demonstrates curcumin's potential to mitigate liver damage and systemic inflammation, suggesting SIRT1 as a promising therapeutic target for heatstroke‐related hepatic injury.

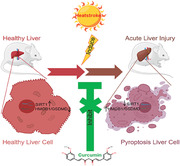

## 1 Introduction

Heatstroke (HS) is an acute condition marked by a core body temperature of ≥42 °C, impacting various organs and systems. Its onset is rapid, with the potential for swift progression and a significant threat to life. The liver, as a central metabolic organ in the human body, is particularly vulnerable to damage during episodes of heat stress. Liver failure resulting from HS is a critical factor in determining the prognosis of individuals afflicted with this condition.^[^ ^1^ ^]^ Hepatocyte necrosis and nuclear collapse have been observed in heatstroke rats in animal model studies.^[^ ^2^ ^]^ Although heatstroke is recognized to induce liver injury, the exact mechanisms remain unclear, necessitating further research for clarification.

Sirtuins, NAD^+^‐dependent deacetylases, are key regulators in cellular processes by removing acetyl groups from lysine residues. They are essential in biological processes such as cell fate, gene regulation, cell cycle, apoptosis, inflammation, and metabolism.^[^ ^3^, ^4^, ^5^ ^]^ Sirtuins play a crucial role in various cellular processes, such as cell differentiation, transcriptional regulation, cell cycle progression, apoptosis, inflammatory responses, metabolic regulation, neuro and cardiovascular physiology, and the initiation and advancement of cancer.^[^ ^6^ ^]^ The broad range of functions exhibited by sirtuins has positioned them as a prominent subject of interest in contemporary biomedical research, with their potential as innovative therapeutic targets for a variety of diseases being widely acknowledged.^[^ ^4^ ^]^ Sirtuin 1 (SIRT1), a particularly conserved member of this family, functions as an NAD^+^‐dependent deacetylase.^[^ ^5^ ^]^ The hepatocellular injury caused by an excessive intake of acetaminophen results in a reduction in the activity of SIRT1 and heme oxygenase‐1, leading to heightened inflammatory responses and elevated levels of oxidative stress.^[^ ^6^ ^]^ In a separate study, the deletion of the SIRT1 gene markedly worsened the chronic tubular injury, tubulointerstitial inflammation, and fibrosis induced by repeated administration of low doses of cisplatin.^[^ ^7^ ^]^ Furthermore, mice that were subjected to heat stress pretreatment exhibited a reduction in SIRT1 levels within mouse lung epithelial‐2 cells.^[^ ^8^, ^9^ ^]^ However, the potential impact of HS on SIRT1 levels in liver tissue and its correlation with HS‐induced liver damage remains uncertain. Additional research is required to elucidate this relationship. Therefore, exploring the association between HS and SIRT1 may offer novel insights into the mechanisms underlying liver damage in HS and present new avenues for therapeutic interventions.

Curcumin, a polyphenolic compound from turmeric (Curcuma longa), is noted for its anti‐inflammatory, antioxidant, lipid‐regulating, and potential antiviral and anticancer properties in medical research.^[^ ^10^ ^]^ Due to its demonstrated efficacy in mitigating HS‐induced kidney injury, curcumin emerges as a promising agent for the prophylaxis and treatment of HS.^[^ ^11^ ^]^ Moreover, studies have shown that curcumin can effectively regulate SIRT1 activity in various disease models and is recognized as a natural activator of SIRT1.^[^ ^12^, ^13^, ^14^ ^]^

These findings indicate that curcumin has the potential to mitigate liver damage caused by heatstroke through the activation of SIRT1. Therefore, this study aims to explore the correlation between SIRT1 and heatstroke‐induced liver injury, as well as to examine the potential mechanism through which curcumin mitigates liver damage resulting from heatstroke. This research seeks to elucidate the involvement of SIRT1 in liver injury during heatstroke and evaluate the efficacy of curcumin as a potential therapeutic intervention for preventing and treating heatstroke‐induced liver damage.

## 2 Results

### 2.1 HS Significantly Induced Liver Injury, Whereas Curcumin Effectively Reversed This Change

**Figure 1:**
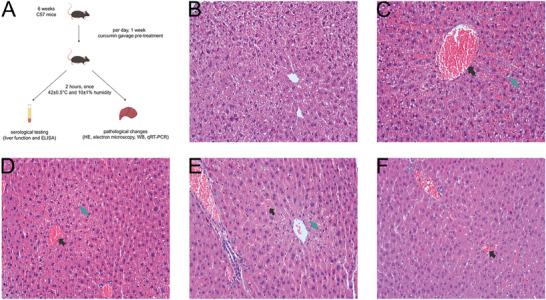
Experiment design and pathological changes. Structural alterations in liver tissue were examined using hematoxylin and eosin (H&E)‐stained sections under optical microscopy (×200). A) Laboratory animal handling flowchart. B) NTT group. C) HTT group. D) H50c group. E) H100c group. F) H200c group. Black arrows indicate congestion, and green arrows indicate cellular vacuolation.

An optical microscope was utilized to examine the structural alterations in hepatocytes within each group (**Figure** **1**). Our findings revealed that, in comparison to the NTT group, the hepatic lobules of the HTT group exhibited marked dilation and congestion of the central veins, along with disorganized hepatic cord structures (Figure 1B,C). Furthermore, significant vacuolation was observed in the hepatocytes surrounding the central veins of the hepatic lobules, accompanied by extensive congestion in the hepatic sinusoids, and eosinophilic changes in hepatocytes could be seen. In comparison to the HTT group, the curcumin pretreatment group exhibited a gradual reduction in both congestion levels and hepatocyte vacuolation as curcumin concentrations increased, accompanied by a progressively clearer delineation of hepatic cord contours (Figure 1C–F).

### 2.2 Curcumin Pretreatment Effectively Prevented Significant Alterations in Hepatocyte Ultrastructure under HS

**Figure 2:**
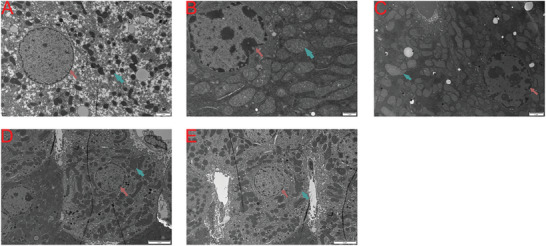
Ultrastructural changes in liver tissue across different groups were investigated using electron microscopy. A) NTT group. B) HTT group. C) H50c group. D) H100c group. E) H200c group. Red arrows indicate the cell nucleus, and green arrows indicate mitochondria.

We utilized electron microscopy to examine the ultrastructural changes in the liver tissue of each group (**Figure** **2**). Compared to the NTT group (Figure 2A), the cytoplasm in the HTT group (Figure 2B) was significantly condensed, the nuclear membrane was markedly indented, and a large amount of heterochromatin was present within the nucleus. The chromatin was significantly aggregated at the nuclear periphery, and parts of the endoplasmic reticulum surrounding the nucleus appeared blurred. The mitochondria were significantly swollen, with some mitochondrial cristae becoming indistinct or disappearing. In contrast to the HTT group, the hepatocytes in the curcumin pretreatment group showed a reduction in intranuclear heterochromatin, some restoration of nuclear morphology, decreased mitochondrial swelling, and looser cytoplasm. This suggests that as the concentration of curcumin pretreatment increased, the liver damage caused by HS was further mitigated (Figure 2B–E).

### 2.3 HS Resulted in Increased Levels of Inflammation and Oxidative Stress in the Serum, Which Were Successfully Attenuated by Pretreatment with Curcumin

**Figure 3:**
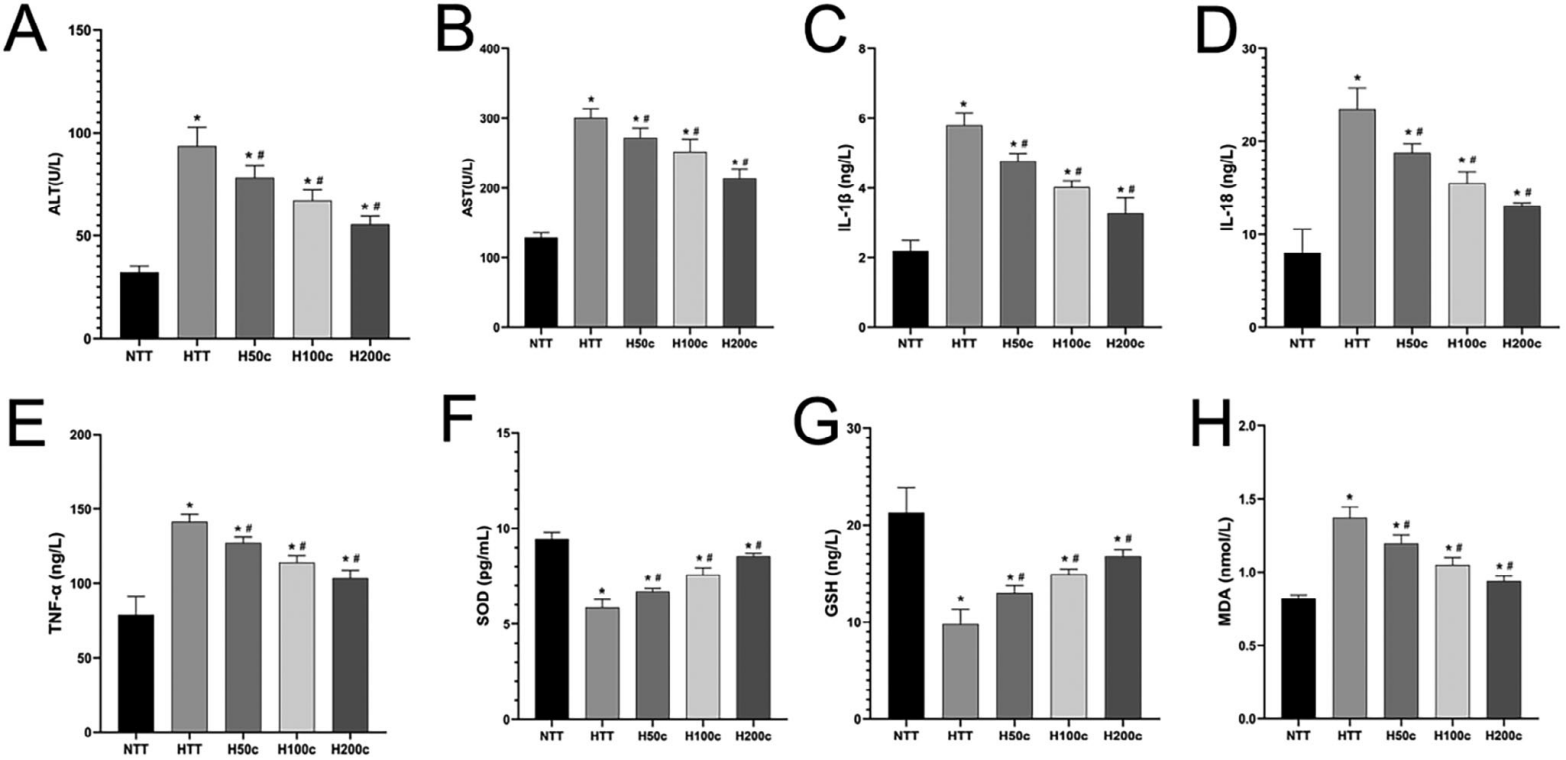
Assessment of serological indices. A,B) Correspond to liver function, as indicated by alanine aminotransferase (ALT) and aspartate aminotransferase (AST), respectively. C–E) Represent the levels of circulating inflammatory markers, including interleukin‐1β (IL‐1β), interleukin‐18 (IL‐18), and tumor necrosis factor‐α (TNF‐α). F–H) Indicative of circulating oxidative stress levels, as measured by superoxide dismutase (SOD), reduced glutathione (GSH), and malondialdehyde (MDA), respectively. *p < 0.05 compared to the NTT group. #*p* < 0.05 compared to the HTT group.

In our study, an automatic biochemical analyzer was used to measure elevated serum ALT and AST levels in HS mice, indicating liver damage (*P* < 0.05; **Figure** **3**A,B). Curcumin pretreatment significantly reduced these levels, suggesting a protective effect against HS‐induced liver injury, with a concentration‐dependent benefit (*P* < 0.05; Figure 3A,B). ELISA analysis showed increased inflammatory and oxidative stress markers (IL‐1β, IL‐18, TNF‐α, MDA) in HS mice, which were significantly lowered by curcumin pretreatment (*P* < 0.05; Figure 3C–E,H). Conversely, antioxidant markers (SOD, GSH) were reduced by HS but increased with curcumin pretreatment, indicating an enhanced antioxidant response (*P* < 0.05; Figure 3F,G). Collectively, these findings indicate that curcumin may mitigate HS‐induced liver injury by reducing inflammation and oxidative stress while boosting antioxidant defenses.

### 2.4 HS Reduced SIRT1 Expression and Increased Liver Injury‐Related Proteins, But High Doses of Curcumin Reversed These Effects

**Figure 4:**
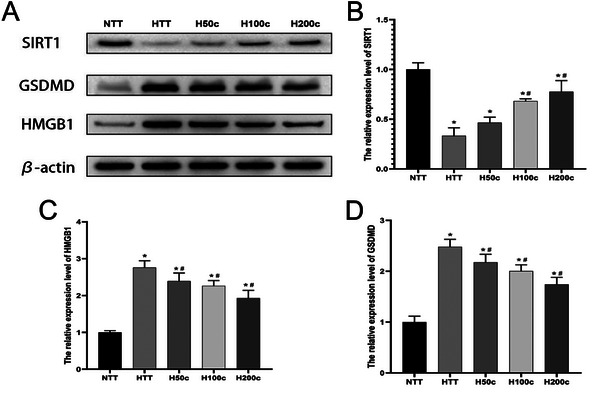
Western blot to assess the relative expression level of SIRT1, GSDMD, and cytoplasmic HMGB1 in liver tissue. A) Representative bands of western blot of liver tissue protein with SIRT1, GSDMD, HMGB1 antibodies. B–D) Relative SIRT1, GSDMD, HMGB1 expression. β‐actin was served as an internal control for protein loading. *p < 0.05 compared to the NTT group. #*p* < 0.05 compared to the HTT group.

Our WB analysis revealed that HS significantly increased GSDMD and HMGB1 protein expression in liver tissue (*P* < 0.05; **Figure** **4**C,D), indicating a role in inflammation and cell death. Curcumin treatment significantly reduced these levels (*P* < 0.05; Figure 4C,D), suggesting an anti‐inflammatory effect. Additionally, HS decreased SIRT1 protein expression (*P* < 0.05; Figure 4B), which is associated with anti‐inflammatory and antioxidant defenses. Curcumin, particularly at higher concentrations, significantly increased SIRT1 expression (*P* < 0.05; Figure 4B), indicating a protective role against HS‐induced liver injury. These results underscore the potential of curcumin in modulating the liver's response to HS.

### 2.5 HS Decreased SIRT1 mRNA Expression and Increased Liver Injury‐Related Protein mRNA Levels, But High Doses of Curcumin Reversed These Effects

**Figure 5:**
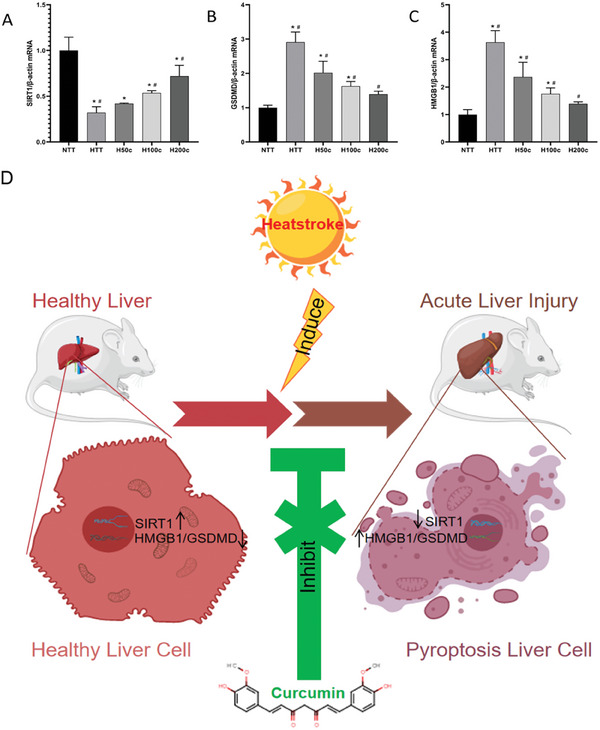
Expression of A) SIRT1, B) GSDMD, and C) HMGB1 mRNA level in liver tissue. β‐actin was served as an internal control for protein loading. *p < 0.05 compared to the NTT group. #*p* < 0.05 compared to the HTT group. D) The mechanism diagram of curcumin in relieving heat stroke.

Our study used qRT‐PCR to analyze liver tissue protein expression patterns post‐HS (**Figure** **5**A–C). We found that HS significantly increased GSDMD and HMGB1 mRNA levels (*P* < 0.05, Figure 5B,C), indicating their roles in liver inflammation and cell death. Curcumin pretreatment reduced these levels (*P* < 0.05, Figure 5B,C), suggesting it can dampen HS‐induced inflammation. Additionally, HS decreased SIRT1 mRNA expression (*P* < 0.05, Figure 5A), linked to anti‐inflammatory processes. Curcumin‐pretreated groups (H100c and H200c), however, showed increased SIRT1 levels (*P* < 0.05, Figure 5A), implying a protective effect against HS‐induced liver injury. These findings highlight curcumin's potential in mitigating HS‐induced liver inflammation and damage. Figure 5D shows the mechanism diagram of curcumin in relieving heat stroke.

## 3 Discussion

Prior research has identified multiple hepatic injury models linked to HS,^[^ ^15^, ^16^, ^17^, ^18^ ^]^ primarily in environments with normal or high humidity levels. This current study aimed to replicate heatstroke in a dry heat setting and observed that liver cell vacuolation, eosinophilic changes in hepatocytes, and sinusoidal congestion and dilation were the primary changes noted in this particular environment. In comparison, the murine model of heat stress utilized in this study demonstrated heightened sinusoidal dilation and congestion, potentially attributed to the prolonged duration of the model and decreased humidity levels. Examination through electron microscopy unveiled a denser distribution of mitochondria within hepatocytes of the HS mice, alongside notable mitochondrial swelling, suggesting potential correlations with intracellular fluid loss and modifications in cell and mitochondrial membrane permeability induced by HS. By utilizing HE staining on liver tissue samples, our study revealed that the HTT group displayed widespread hepatocyte edema and vacuolar liver cells arranged in a radial pattern around the central vein, in contrast to the NTT group. This observation suggests a potential link between the more pronounced ischemia and hypoxia near the central veins of the lobules, induced by HS‐induced blood flow stasis. Furthermore, treatment with curcumin in varying concentrations showed a gradual improvement in the histological alterations mentioned above.

Prior research has demonstrated the efficacy of curcumin in mitigating heat stress‐induced kidney injury^[^ ^10^ ^]^ and myocardial injury.^[^ ^19^ ^]^ In the current investigation, it was observed that elevated doses of curcumin (100/200 mg kg d^−1^) resulted in a notable upregulation of SIRT1 protein and SIRT1 mRNA expression in liver tissue, concomitant with a decrease in the severity of heat stress‐induced liver damage. SIRT1, a significant protein deacetylase, has been identified as playing a crucial role in various diseases and is regarded as a protective element. Deletion of the SIRT1 gene has been shown to worsen liver ischemia‐reperfusion injury.^[^ ^20^, ^21^, ^22^ ^]^ The upregulation of SIRT1 expression effectively alleviated lipopolysaccharide‐induced oxidative stress and liver injury.^[^ ^23^, ^24^, ^25^, ^26^ ^]^ Through the analysis of serum levels of GSH, SOD, and MDA, it was observed that the rise in GSH and SOD levels exhibited a direct correlation with the concentration of curcumin. Conversely, the levels of MDA decreased as the concentration of curcumin increased. These findings suggest that curcumin may effectively mitigate the heightened oxidative stress induced by HS. Consequently, targeting SIRT1 could offer a promising therapeutic approach for acute liver injury caused by HS, with curcumin demonstrating potential as an efficacious treatment for preventing such damage.

The cytosolic protein GSDMD, encoded by the GSDMD gene within the Gasdermin family, serves as the primary mediator of pyroptosis.^[^ ^21^ ^]^ Our research demonstrates that HS significantly enhances the expression of GSDMD in hepatocytes, a process that can be mitigated by the administration of curcumin. Additionally, this intervention led to an elevation in SIRT1 expression levels. In the context of pyroptosis‐induced activation, IL‐1β and IL‐18 are released into the extracellular space concurrent with GSDMD activation, contributing to the escalation of the inflammatory response.^[^ ^22^ ^]^ Our investigation quantified serum levels of IL‐1β and IL‐18, revealing a significant increase following exposure to HS, correlating with the severity of liver damage and the expression of GSDMD in hepatic tissue. Therefore, it is hypothesized that the conventional pyroptosis theory may be applicable to the heat stress model under consideration. Upon initiation of pyroptosis, pro‐inflammatory cytokines such as IL‐1β and IL‐18 are released into the extracellular environment before entering the circulation, contributing to the systemic inflammatory response associated with the progression of thermotoxic shock and subsequent multiple organ dysfunction in HS.^[^ ^27^, ^28^, ^29^, ^30^ ^]^

HMGB1 is a non‐histone chromatin‐associated protein found in eukaryotic cells that can be released into the extracellular space in response to external stimuli.^[^ ^31^ ^]^ It functions as a damage‐associated molecular pattern (DAMP) and plays a role in various inflammatory response processes.^[^ ^32^, ^33^ ^]^ Post‐translational modifications, such as acetylation, phosphorylation, and methylation, are crucial regulatory mechanisms for the nuclear‐cytoplasmic translocation of HMGB1, ultimately impacting the activation of downstream signaling pathways.^[^ ^34^, ^35^ ^]^

Furthermore, it has been observed that pyroptosis facilitated by GSDMD‐N can result in the passive release of pro‐inflammatory cytokines, such as HMGB1, into the extracellular environment, thereby exacerbating the inflammatory response.^[^ ^36^ ^]^ Our investigation involved the assessment of HMGB1 expression within hepatocyte cytoplasm, revealing that HS effectively triggered an upregulation of HMGB1 expression, a response that was attenuated by the administration of curcumin. The concurrent reduction in HMGB1 expression and the elevation of SIRT1 expression levels suggest that curcumin may mitigate the inflammatory response by modulating the expression of both HMGB1 and SIRT1.

Our study results highlight that heat stress is believed to increase HMGB1 expression and its acetylated translocation, promoting GSDMD activation and extracellular release, which worsens liver injury. Additionally, curcumin demonstrates efficacy in reducing oxidative stress and tissue damage, lowering HMGB1 and GSDMD expression, and is associated with increased SIRT1 levels. SIRT1 is recognized as a pivotal protein with the ability to mitigate oxidative stress and inflammatory reactions in numerous diseases.^[^ ^37^ ^]^ Previous research has identified curcumin as a potent stimulator of SIRT1 and SIRT3.^[^ ^38^ ^]^ It is hypothesized that curcumin may impede oxidative stress by upregulating SIRT1 expression levels and potentially hinder GSDMD activation by restricting HMGB1 translocation through acetylation suppression. Considering the multifaceted nature of curcumin's actions,^[^ ^39^, ^40^ ^]^ the mitigation of liver damage caused by HS extends beyond merely the direct suppression of oxidative stress and inflammatory processes. The therapeutic impact of curcumin is likely due to its ability to modulate the expression levels of critical proteins like GSDMD and SIRT1, which are integral to the body's response to cellular stress. Curcumin's protective effect on the liver may thus be exerted through a dual mechanism: it diminishes immediate oxidative and inflammatory reactions and simultaneously affects an extensive network of molecular pathways that are crucial for maintaining liver health and functionality.

Our study indicates that curcumin pretreatment significantly mitigates the severity of acute liver injury induced by HS, with higher concentrations proving more effective by decreasing pyroptosis‐related proteins and upregulating SIRT1. This implicates pyroptosis in HS‐induced liver injury and positions SIRT1 as a potential target for curcumin's protective effects. We observed that elevated SIRT1 levels contribute to the amelioration of liver injury. Curcumin's multifaceted biological activities likely underpin its benefits. We intend to refine our experiments to further elucidate the mechanisms of HS‐induced liver injury and curcumin's protective role, to discover novel therapeutic strategies for managing HS‐induced liver injury and enhancing patient outcomes.

Previous studies have shown that curcumin has different routes and doses of administration and pathways of action in cellular or animal models of different diseases. For instance, curcumin was administered at doses of 10 × 10^−6^, 20 × 10^−6^, and 40  × 10^−6^ m to U251 glioblastoma cells. The highest dose of 40  × 10^−6^ m was found to significantly induce oxidative stress, inflammation, and apoptosis, highlighting a dose‐dependent inhibitory effect on cell proliferation and migration.^[^ ^41^ ^]^ This aligns with our findings, where higher doses of curcumin (200 mg kg^−1^) offered optimal protection against liver injury in the heatstroke model. Curcumin's role in ischemia‐reperfusion (I/R) injury models was also investigated,^[^ ^42^ ^]^ where curcumin at 100 mg kg^−1^, administered intraperitoneally, effectively reduced renal and liver injury markers. The study demonstrated that curcumin significantly decreased oxidative stress and apoptotic pathways, consistent with the benefits observed in our study through SIRT1 upregulation and attenuation of HS‐induced liver damage. In another study on neurodegenerative damage induced by amyloid beta (Aβ1‐42),^[^ ^43^ ^]^ curcumin's neuroprotective effects were assessed in synaptosomes treated with 5 µg mL^−1^ curcumin. This treatment demonstrated a reduction in oxidative stress and neuroapoptosis, supporting curcumin's antioxidant properties across different tissues and models. Additionally, research on the co‐administration of boric acid and curcumin found that curcumin enhances synaptosomal integrity and protects against oxidative and nitrosative stress in neurodegenerative models. This further emphasizes curcumin's broad‐spectrum therapeutic potential, particularly in conditions involving oxidative stress. Our study's oral gavage administration contrasts with other methods, such as intraperitoneal injections used in renal and liver I/R injury models,^[^ ^44^ ^]^ suggesting that the route of administration may influence curcumin's bioavailability and therapeutic efficacy. For instance, other researchers observed that intraperitoneal administration achieved substantial effects on reducing oxidative stress and modulating apoptotic pathways at lower doses compared to our higher oral doses. In summary, the comparative analysis underscores the versatility of curcumin as a therapeutic agent across various conditions and administration routes. The findings from our study, alongside those from other models, suggest that curcumin's efficacy is dose‐dependent and route‐specific, making it a promising candidate for mitigating heatstroke‐induced liver injury and other oxidative stress‐related conditions.

## 4 Conclusion

Our study indicates that curcumin pretreatment significantly mitigates the severity of acute liver injury induced by HS, with higher concentrations proving more effective by decreasing pyroptosis‐related proteins and upregulating SIRT1. This implicates pyroptosis in HS‐induced liver injury and positions SIRT1 as a potential target for curcumin's protective effects. We observed that elevated SIRT1 levels contribute to the amelioration of liver injury. Curcumin's multifaceted biological activities likely underpin its benefits. We intend to refine our experiments to further elucidate the mechanisms of HS‐induced liver injury and curcumin's protective role, to discover novel therapeutic strategies for managing HS‐induced liver injury and enhancing patient outcomes.

## 5 Experimental Section

### Animals

In this study, 50 SPF‐grade C57BL/6 mice aged 6 weeks and weighing 22–25 g were carefully selected to ensure experimental accuracy and reliability. These mice were sourced from the Experimental Animal Center of Xinjiang Medical University, ensuring high‐quality subjects for the research. Mice were randomly assigned to five groups (10 per group) for balanced experimentation. They were housed in SPF facilities at the Experiment Animal Department of Xinjiang Military Region General Hospital, with controlled environmental conditions: a constant temperature of 22 ± 2 °C, 20–30% humidity, and a 12 h light/dark cycle. Initially, mice had unrestricted access to standard chow and water to acclimate and maintain health. After a 2 week adaptation period, they received experimental pretreatment. The study was conducted in strict accordance with ethical standards for laboratory animal care. Approval was obtained from the Ethics Committee of Xinjiang Military Region General Hospital (Approval No. DWLL20200111), complying with the National Institutes of Health guidelines for animal welfare, emphasizing both the ethics and scientific integrity of the research.

### Curcumin Pretreatment, Establishment of the HS Mouse Model, and Sample Collection

In this experimental design, 50 mice were meticulously selected and randomly assigned them into five distinct groups, each consisting of 10 mice. The groups were as follows: normal temperature control (NTT), high temperature control (HTT), and three HS groups pretreated with different concentrations of curcumin (H50c, H100c, H200c). Curcumin was prepared as a suspension using 0.5% sodium carboxymethyl cellulose solution as the vehicle. The selected concentrations of curcumin were based on previous studies indicating its potential role in preventing and treating HS. Specifically, mice in the H50c, H100c, and H200c groups received by gavage at doses of curcumin at 50, 100, or 200 mg kg per day, respectively.^[^ ^10^, ^12^, ^13^ ^]^ In the study, control mice received no pretreatment, while curcumin‐pretreated groups were given oral curcumin daily for seven days to assess its protective effects against heat stroke (HS) in mice. The aim was to explore curcumin's therapeutic mechanisms and provide a foundation for clinical use. At the simulated Climate Cabin for the Special Environment of Northwest China, a heatstroke (HS) model was developed with precise temperature and humidity control. Mice were exposed to 42 ± 0.5 °C and 10 ± 1% humidity for 2 h to induce HS symptoms. Postexposure, they were anesthetized with sodium pentobarbital for safe blood collection and liver sampling. Samples were preserved in liquid nitrogen for molecular analysis. All procedures were conducted ethically, with euthanasia by cervical dislocation, adhering to animal welfare standards and ensuring valuable HS research data. The laboratory animal handling flowchart of the experiment is shown in Figure 1A.

### Preparation of Histopathology Slides of Liver Tissue

To explore pathological liver changes in mice, we processed samples through a detailed histology protocol, which was described before.^[^ ^45^, ^46^, ^47^ ^]^ Briefly, liver tissues were fixed in formalin for 2 weeks to preserve structure and morphology, then dehydrated in graded alcohol before paraffin embedding. Tissue sections were cut and stained with H&E to highlight cellular details. Microscopic examination revealed the microstructure, aiding the understanding of heatstroke's effects on the liver and guiding molecular research.

### Examining Minute Alterations in Liver Tissue Using an Electron Microscope

To study the microstructure of mouse livers, detailed electron microscopy sample preparation was performed according to the protocol.^[^ ^48^, ^49^, ^50^ ^]^ Liver samples were cut into 2 mm sections and fixed in 2.5% glutaraldehyde in 0.1 m phosphate buffer at 4 °C for 2 weeks. After washing with phosphate buffer, samples were post‐fixed with 1% osmium tetroxide for 1 h. Dehydration followed using graded ethanol solutions and acetone. Samples were then infiltrated with Spurr's resin at room temperature, initially at a 1:1 ratio for one hour, then at a 1:3 ratio overnight. After infiltration, samples were polymerized at 70 °C for 9 h. Finally, samples were stained with uranyl acetate and lead citrate to enhance contrast and examined under a transmission electron microscope to visualize the ultrastructural effects of heatstroke on liver cells and tissues.

### Collection of Serum Samples and Detection of Associated Biomarkers

Prior to analysis, mouse blood samples were incubated for 2 h to ensure thorough separation of cells and serum. Post‐incubation, centrifugation at 3000 rpm for 15 min isolates the serum, which was then stored at −20 °C to preserve bioactivity. Liver function was assessed using a Mindray BS‐180 analyzer to measure ALT and AST serum levels, indicators of liver cell damage. Elevated levels suggest HS‐induced liver injury. Serum levels of key antioxidants GSH (catalog: F2658‐A, Fankew) and SOD (catalog: F2389‐A, Fankew), alongside the lipid peroxidation marker MDA (catalog: F9264‐A, Fankew), were precisely quantified using enzyme‐linked immunosorbent assay (ELISA) kits. This comprehensive assessment elucidated the dynamics of oxidative stress and the integrity of cellular membranes in response to HS, shedding light on the body's endogenous antioxidant mechanisms and the degree of oxidative damage sustained. Concurrently, the ELISA technique was employed to measure inflammatory cytokines IL‐1β (catalog: F2040‐A, Fankew), IL‐18 (catalog: F2169‐A, Fankew), and TNF‐α (catalog: F2132‐A, Fankew), which are pivotal in deciphering the systemic inflammatory cascade activated by HS. These measurements provided a nuanced view of the inflammatory processes at play. The integration of these ELISA‐based metrics—encompassing oxidative stress, lipid peroxidation, and inflammatory responses—enabled us to delineate a multifaceted physiological profile. This profile captures the intricate interplay and broad impact of HS on liver function and overall health, thereby enriching our comprehension of the pathophysiological sequelae of HS and informing potential therapeutic interventions.

### Preparation of Protein Samples and Detection of Protein Content through Western Blot (WB) Analysis

Western blotting was integral to our study, with tissue samples homogenized and lysed on ice, followed by centrifugation to isolate soluble proteins. Protein concentrations were determined using a BCA assay kit (catalog: P0012, Beyotime), standardized to 2 µg µL^−1^, and prepared for analysis by heating and storage at −20 °C. SDS‐PAGE (catalog: P1200, Solarbio) was conducted for protein separation, and proteins were transferred to PVDF membranes (catalog: IPVH00010, Solarbio), blocked, and incubated with primary and secondary antibodies. Protein bands were visualized using BeyoECL Plus (catalog: P0018S, Beyotime) and analyzed with the ChemiDoc MP system and Image Lab software, ensuring optimal exposure for accurate quantification of protein expression levels, revealing molecular changes due to heat stress in liver tissues. Primary antibodies were used against SIRT1 (catalog: 3931S, Cell Signaling Technology), HMGB1 (catalog: AG2167, Beyotime), and gasdermin D (GSDMD) (catalog: ab219800, Abcam), and a secondary antibody conjugated to HRP (catalog: ab205718, Abcam) (all diluted 1:5000 in TBST). The ChemiDoc MP system and Image J software facilitated imaging and quantification, allowing us to precisely assess target protein expression and elucidate heatstroke's impact on liver tissue.

Extraction of tissue mRNA and quantification of mRNA expression levels using quantitative reverse‐transcriptase polymerase chain reaction (qRT‐PCR) assay: The liver tissue was homogenized using TRIzol reagent (catalog: 15596026CN, Thermo Fisher) followed by reverse transcription of the total RNA into complementary cDNA utilizing the RevertAid First Strand cDNA Synthesis Kit (catalog: k1622, Thermo Fisher) and a PCR instrument (Thermo Fisher C1000 touch; Shanghai Thermo Fisher Scientific Co., Ltd.). Following this, the quantitative PCR reaction system should be prepared utilizing SGExcel FastSYBR Mixture (catalog: B532955, Sangon Biotech), β‐actin:5′‐ GATGGTGGGAATGGGTCAGAAGG‐3′(sense) and 5′‐ TTGTAGAAGGTGTGGTGCCAGATC‐3′ (antisense) or HMGB1: 5′‐ AAGGCTGACAAGGCTCGTTATG‐3′(sense) and 5′‐ GGCGGTACTCAGAACAGAACAAG‐3′ (antisense) or GSDMD: 5′‐ CTCCAGAACCAGGCACCTTCAG‐3′(sense) and 5′‐ AGCACCTCGGTCACCACAAAC‐3′ (antisense) or SIRT1: 5′‐ GCACATGCCAGAGTCCAAGTTTAG‐3′(sense) and 5′‐ ATCCAGATCCTCCAGCACATTCG‐3′ (antisense), and the cDNA template. The real‐time quantitative PCR reactions and detections were conducted utilizing a Jielaimei QX600 real‐time fluorescence quantitative PCR instrument (Sichuan Jielamei Technology Co., Ltd.) with a two‐step amplification protocol of 35 cycles (denaturation at 95 °C for 10 s; annealing/extension at 60 °C for 30 s). The 2^‐ΔΔCT method was used to assess relative mRNA expression according to the method protocol,^[^ ^15^, ^18^, ^27^ ^]^ normalizing target gene levels to the housekeeping gene β‐actin to control for sample variability. This normalization ensured the reliability and comparability of these results, providing valuable insights into gene expression changes under various conditions.

### Statistical Analysis

The data are presented as mean ± standard deviation (*n* = 10) for each group. Prior to analysis, normality tests were performed using the Shapiro‐Wilk test to determine the distribution of the data. For normally distributed data, one‐way analysis of variance (ANOVA) was used to assess inter‐group differences, followed by the least significant difference (LSD) test for post‐hoc multiple comparisons. For non‐normally distributed data, non‐parametric methods, such as the Kruskal‐Wallis test followed by Dunn's post‐hoc test, were employed. Statistical analyses were conducted using SPSS software (version 27.0; IBM Corp.), with a *P*‐value of less than 0.05 considered statistically significant.

## Conflict of Interest

The authors declare no conflict of interest.

## Data Availability

The data that support the findings of this study are available from the corresponding author upon reasonable request.
